# Ætiology and Treatment of Pneumonia

**Published:** 1875-03-15

**Authors:** 


### ÆTIOLOGY AND TREATMENT OF PNEUMONIA.

### DISCUSSION OF NEW YORK ACADEMY OF MEDICINE, FEBRUARY 4, 1875.

*From New York Medical Journal, March*, 1875.

THERE was a large gathering at the Academy to hear the discussion on the "Ætiology and Treatment of Pneumonia."

Dr. Austin Flint, by request of the President, opened the discussion by indicating some points of interest that might be freely considered. He noted first the diverse characteristics of the disease at different localities, and at different times in the same localities. In the Southern States pneumonia has always been a more grave disease than it has been in the North. Again, the disease as it manifests itself at the present time in this city is of a more dangerous character than we are usually in the habit of considering it. Dr. Flint gave, in this connection, the brief histories of several cases which came under his care during the month of January, each of them dying in a few days. As regarded the treatment of the disease, he took up fully the use of bleeding as a therapeutical agent, and considered the views and results of Louis on this matter. Louis reported seventy-eight cases in which there were twenty-eight deaths; he reported also other twenty-nine cases, having four deaths. In his observations he did not regard the subject of mortality, but simply whether bleeding did or did not have a tendency to shorten the disease. The conclusions that he arrived at were, that if bleeding were practiced in the earlier days of the disease, it not only had a happy effect, but shortened its duration by four or five days; whereas, if the bleeding were delayed till late in the disease, no good effect was obtained. Jackson reported fifty-one cases in which he had eight deaths.

His treatment was the same as that of Louis. Dr. Flint reported one hundred and thirty-three cases that came under his own observation, and in which bleeding was practiced only in twelve of them. There were thirty-five deaths, but a point of interest connected with them was that they were observed in different cities: sixty-four at Buffalo, with eleven deaths; eleven at Louisville, with seven deaths; fifty-eight at New Orleans, with seventeen deaths. He said also that there was one very important thing to bear in mind in the consideration of pneumonia; and that was that when the inflammation was confined to a single lobe, and there were no complications, the tendency was to recover; and as regards the treatment, whether bleeding exerted an influence in preventing the complications which might arise, such as gangrene of the lungs, extension of the inflammation, pericarditis, and heart-clot. He referred also to the value of opium in diminishing the constitutional disturbance; and to the plan presented latterly, of keeping the heat of the patient down by antipyretic treatment. He was in favor of the administration of quinia, and had seen marked benefit from its use. Dr. Flint read a short description from a medical periodical of pneumonia developing in a high school which had been exposed to the influence of sewer-gas. The facts were as follows: The sewer authorities, in defiance of the protest of the principal of the school, placed a ventilator so as to infect the institution with sewer-gas. Shortly after a number of cases of pneumonia developed, and, as a consequence, the school had to be closed. For many years previous to this there had been no sickneas in the institution, and, when the ventilator was removed, no new cases occurred, leaving the strong inference that the sewer-gas was the peccant agent.

Dr. W. H. Thomson read the histories of five cases of pneumonia which had recently been under observation in Bellevue Hospital, and in which the main treatment had been antipyretic. The method pursued was to apply to the chest ice-bags till the temperature fell. Of the five cases only one proved fatal. Dr. Thomson read a selection from Niemeyer bearing out his opinions as to the effect of cold in keeping down the temperature and improving the chances of the patient. He said also that the medicinal treatment consisted in the administration of ten grains of the carbonate of ammonia every two hours, with one grain of quinia every hour.

Dr. A. L. Loomis said that in the consideration of pneumonia the two main elements to bear in mind were the ætiology and treatment; and of the causes the most important was age. We saw it mostly between twenty and forty years, and again after sixty—the pneumonia of children was broncho-pneumonia. The predisposing cause was the cause of the disease, and by the predisposing cause was meant anything that lowers the vitality of the patient. Here in New York we are all staggering under the influence of malaria, and with any slight exposure there may follow a pneumonia. Inanition, the continual use of alcoholics, the poison of sewer-gas, septicæmia, rheumatism, pyæmia, and all those conditions which debilitate, render the system at any time liable to an attack from a cause which, in sound health, would prove inoperative. Dr. Loomis said also that, in his experience, idiopathic pneumonia was of such rare occurrence that he questioned its existence. He did not think there could be much difference of opinion as to the injury received from venesection in pneumonia, where the predisposing cause was debility; the indication was to find and combat the cause of the debility. As to the use of ice in the treatment of the disease his opinion was decidedly against it, though he was sorry he had not a more extensive experience. He found that it kept down the temperature as long as it was applied to the chest, but he found also that at the same time there was a tendency for the disease to spread. He found, on coming on duty at the Mount Sinai Hospital, several cases treated by ice, but they all died. The cause of the high temperature was the metamorphosis of tissue that takes place, and the remedy, in his experience, that prevented this change was the sulphate of quinia. He had found a full dose of the drug to lower the temperature from one to one and a half degrees, and considered that ten grains should be given at a dose. When the heart began to flag and grow weak the remedy was alcohol. He found it the only thing to be relied on to stimulate the heart to renewed activity, and ward off œdema of the lungs and other causes of failure of the circulation.

